# Expression of cytokines in gingival crevicular fluid associated with tooth movement induced by aligners: a pilot study

**DOI:** 10.1590/2177-6709.23.5.041-046.oar

**Published:** 2018

**Authors:** Vitória de Oliveira Chami, Livia Nunes, Jonas Capelli

**Affiliations:** 1 Universidade Federal de Santa Maria, Programa de Pós-Graduação em Ciências Odontológicas (Santa Maria/RS, Brazil).; 2 Universidade do Estado do Rio de Janeiro, Programa de Pós-Graduação em Odontologia (Rio de Janeiro/RJ, Brazil).; 3 Universidade do Estado do Rio de Janeiro, Departamento de Odontologia Preventiva e Comunitária (Rio de Janeiro/RJ, Brazil).

**Keywords:** Invisalign, Gingival crevicular fluid, Cytokines

## Abstract

**Introduction:**

The search for more aesthetic and comfortable orthodontic devices has led to an increase in the use of clear aligners.

**Objective:**

To increase knowledge on biological mechanisms of orthodontic tooth movement using Invisalign aligners.

**Methods:**

This study included 11 patients with a mean age of 23.6 ± 4.8 years. Cases planning included alignment and leveling of lower incisors using Invisalign aligners. Gingival crevicular fluid samples were collected from the lower incisors on the day of delivery of aligner number 1 (T0) and after 1 (T24h), 7 (T7d), and 21 (T21d) days. During the observation period of the study, the patients used only the aligner number 1. Levels of nine cytokines were quantified using Luminex’s multi-analysis technology. Non-parametric tests were used for comparisons between cytokine expression levels over time.

**Results:**

Cytokine expression levels remained constant after 21 days of orthodontic activation, except those of MIP-1β, which presented a statistical difference between T24h and T21d with a decrease in the concentration levels. IL-8, GM-CSF, IL-1β, MIP-1β, and TNF-α showed the highest concentrations over time.

**Conclusions:**

The different behavior in the levels of the investigated cytokines indicates a role of these biomarkers in the tissue remodeling induced by Invisalign.

## RESUMO

**Introdução:**

a busca por dispositivos ortodônticos mais estéticos e confortáveis gerou um aumento no uso de alinhadores transparentes.

**Objetivo:**

ampliar o conhecimento sobre os mecanismos biológicos associados ao movimento dentário ortodôntico promovido por alinhadores Invisalign^®^.

**Métodos:**

a amostra foi constituída por 11 pacientes, com idade média de 23,6 ± 4,8 anos. O planejamento dos casos incluiu alinhamento e nivelamento de incisivos inferiores usando os alinhadores. O fluido gengival crevicular foi coletado na superfície vestibular de incisivos inferiores no dia da entrega do alinhador número 1 (T0) e após 1 (T24h), 7 (T7d) e 21 (T21d) dias. Durante o período de observação do estudo, os pacientes utilizaram apenas o alinhador número 1. Os níveis de nove citocinas foram quantificados por meio do sistema Luminex de multianálise. Testes não paramétricos foram realizados para comparações entre os níveis de expressão de citocinas ao longo do tempo.

**Resultados:**

a concentração das citocinas manteve-se constante após 21 dias de ativação ortodôntica, exceto a MIP-1β, que apresentou uma redução estatisticamente significativa entre os tempos T24h e T21d. As IL-8, GM-CSF, IL-1β, MIP-1β e TNF-α apresentaram as maiores concentrações ao longo do tempo.

**Conclusão:**

a constância na expressão dos níveis das citocinas parece estar compatível com o estímulo mecânico induzido por alinhadores.

## INTRODUCTION

The mechanical stimulus associated with induced tooth movement must be performed smoothly and continuously, promoting a series of tissue and cell changes in the periodontium, accompanied by an increased release of inflammatory mediators, such as cytokines.

These biochemical mediators are low molecular weight proteins that are involved in all phases of inflammation.^1^ Proinflammatory cytokines induce the classic characteristics of inflammation, through vasodilation and invasion of tissues by leukocytes, while anti-inflammatory cytokines are involved in the resolution of inflammatory process.

The cytokines are characterized by overlapping and interlocking functions, and provide information on local cell metabolism, reflecting periodontal health status and bone remodeling.^2^ ^,^ ^3^ Thereby, the evaluation of this mediators is important to elucidate the molecular mechanisms that occur during orthodontic movement.

To monitor the biological responses during orthodontic therapy, gingival crevicular fluid (GCF) analysis is the method of choice because it is noninvasive and feasible to collect the fluid at different time intervals without producing sequelae. In addition, cytokine expression in GCF can provide an indirect measurement of changes in the periodontal ligament.^4^ ^,^ ^5^

The Invisalign system induces tooth movement using plastic trays called aligners. In selected cases, these aligners represent an alternative to the traditional metal or ceramic brackets.^6^ They produce intermittent forces on the teeth because they are removable and their strength levels fluctuate over the course of treatment. Kuncio et al^7^ suggested that teeth moved with aligners are not subjected to the typical stages of orthodontic movement as described by Krishnan and Davidovitch.^2^

There are only few studies in the orthodontic literature that have clarified the biological phenomena of orthodontic movement using aligners. Thus, the aim of the present study was to detect and quantify nine cytokines: interleukin (IL)-β, (IL)-7, (IL)-8, (IL)-17, colony stimulating factors G-(CSF), GM-(CSF), monocyte chemoattractant protein(MCP)-1, macrophage inflammatory protein (MIP)-1β, and tumor necrosis factor (TNF)-α, in orthodontically moved teeth using aligners.

## MATERIAL AND METHODS 

### Sample selection

This study sample consisted of 11 patients (6 women [54.5%] and five men [45.5%]) with a mean age of 23.63 ± 4.88 years, undergoing treatment at the Orthodontic Clinic of the Dental School of *Universidade do Estado do Rio de Janeiro*(Brazil).

The inclusion criteria were: full permanent dentition; Little’s Irregularity Index (Little,^8^ 1975) between 3 and 5 mm in the anterior segment of the lower arch; absence of caries or restorations in the anterior teeth; and full-mouth plaque score ≤ 30% and full-mouth bleeding score ≤ 10%. Patients with autoimmune diseases and those who were pregnant or lactating were not eligible. Additionally, participants exhibiting a prolonged use of medication (e.g., antibiotics, antihistamines, cortisone, hormones, and any other medication that may interfere with the inflammatory process or may adversely affect the periodontium) three months before and during the study period were excluded. This study protocol was approved by the Research Ethics Committee of *Hospital Universitário Pedro Ernesto/UERJ*. A signed informed consent was obtained from patients’ parents prior to the study.

### Periodontal control

One week before the start of orthodontic treatment and during collection times, all patients received oral hygiene instructions to demonstrate the correct use of brush and floss to control possible gingival inflammation.

During each day of GCF collection, a periodontal evaluation was performed. Specifically, the visible plaque index (VPI) and bleeding on probing (BOP) were recorded of the buccal, lingual, mesial, and distal aspects of the lower incisors, using a manual periodontal probe, type Goldman-Fox/Williams (Hu-Friedy, Chicago, IL, USA).

### Orthodontic devices

The Invisalign aligner (Align Technology, San Jose, California) was the orthodontic device used in the study. Using the ClinCheck software, patient planning, including alignment and leveling of the anterior teeth, was performed by a single operator. The inferior arch was selected to be evaluated considering that the Irregularity Index of lower incisors^8^ was one of the inclusion criteria.

During the 21-day observation period of the study, the patients used only the number 1 aligner. Before insertion of the aligner, patients were instructed on the use and hygiene of the device. It should be noted that there was no bonding or interproximal reduction in the teeth during this stage of treatment.

### Immunological monitoring

**Figure 1:**
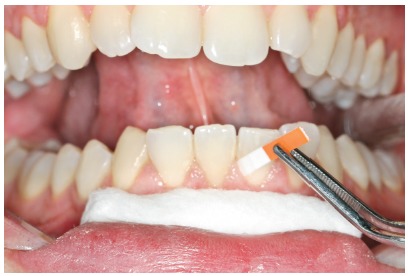
Samples were collected from the vestibular face of lower central incisors and a lateral incisor (teeth #31, #41, #42).

The GCF samples were collected from the vestibular face of lower central incisors and lateral incisor (teeth #31, #41, and #42) at different times: on the day of delivery of the aligner (T0) and at 1 (T24h), 7 (T7d), and 21 (T21d) days after orthodontic activation (Fig 1). 

Prior to GCF collection, the supragingival plaque was carefully removed. The collection sites were isolated using cotton rolls and dried with light air jets. GCF was collected using absorbent paper strips (Periopaper^®^, Interstate Drug Exchange, Amityville, NY, USA), which were inserted 1-2 mm into the gingival sulcus and maintained for 30s. Samples contaminated with blood were discarded, and a new sample was collected a few minutes later from the same site. Paper tips from the same patient were pooled in a sealed Eppendorf plastic tube and stored at -80^o^C until analysis.

Thereafter, the GCF samples were thawed, and biomarkers were detected using Luminex multi-analyte technology (Bio-Plex Pro^TM^ Human Cytokine Grp I Panel 17-Plex, Catalog no. M50-00031YV, BIO-RAD, Hercules, CA, USA) according to the manufacturer’s instruction. Samples were incubated with antibodies immobilized on color-coded microparticles, washed to remove unbound material, and then incubated with biotinylated antibodies to the molecules of interest. After further washing, the streptavidin-phycoerythrin conjugate that binds to the biotinylated antibodies was added before the final washing step. The Luminex analyzer was used to determine the magnitude of the phycoerythrin-derived signal in a microparticle-specific manner.

### Statistical analysis

The Statistical Package for Social Sciences v. 13.0 (SPSS Inc., Chicago, IL, USA) was used for data analysis. The normality of the sample was verified using the Shapiro-Wilk test; therefore, non-parametric tests were selected for analysis. Differences in the expression levels of each mediator over time were evaluated using the Friedman’s test. When significant interactions were observed, a Bonferroni-corrected Wilcoxon paired signed-rank test was used to evaluate the significance of the difference between baseline and the other time points. A p-value of less than 0.05 was considered as statistically significant.

## RESULTS

### Periodontal

**Figure 2:**
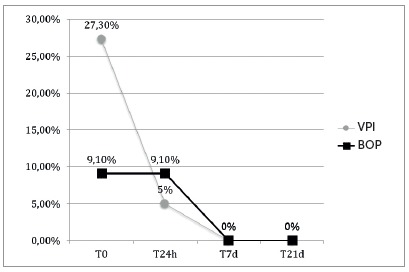
Descriptive analysis of the percentage of sites with visible plaque (VPI) and gingival bleeding (BOP).

During the study period and GCF collection, all patients exhibited a clinically satisfactory oral hygiene. Data collected for the BOP showed values below 30% of the evaluated sites and VPI below 10% (Fig 2). In addition, the evaluated indices decreased over time.

### Inflammatory mediators 

The levels of the different GCF biomarkers are summarized in Table 1.

**Table 1:** Total amount of cytokines throughout the collection times.

Cytokines	T0		T24h		T7d		T21d		P
	Median	interquartile range	Median	interquartile range	Median	interquartile range	Median	interquartile range	
IL-1β	15.23	27.03	11.91	32.46	14.15	23.56	8.55	11.13	0.654
IL-7	0.18	0.63	0.0	0.89	0.34	0.34	0.34	0.34	0.992
IL-8	151.44	249.52	137.16	302.48	85.46	115.37	104.64	48.03	0.138
IL-17	1.29	2.66	2.59	2.61	2.59	2.84	1.91	2.16	0.405
G-CSF	2.05	4.28	2.05	3.99	2.36	4.07	3.28	3.37	0.504
GM-CSF	60.84	15.79	58.69	22.52	56.53	11.98	59.64	30.74	0.921
MCP-1	0.0	1.42	1.04	1.42	0.84	1.04	0.61	1.04	0.981
MIP-1β	10.03	17.77	12.95	13.79	15.45	10.29	10.03	5.44	0.020*
TNF-α	0.78	3.47	1.44	2.22	1.44	3.47	0.78	3.00	0.625

Data are presented as median and interquartile deviations (pg). p: differences over time, p ≤ 0.05 represents a statistical difference.

* significant difference between T24h and T21d.

All cytokines, IL-1β, IL-7, IL-8, IL-17, G-CSF, GM-CSF, MCP-1, MIP-1β, and TNF-α were identified and quantified at all times. The levels of all mediators, except MIP-1β, were similar over time with no significant change. In particular, decreases over time were seen for all these GCF biomarkers.

At the pairwise comparisons, the level of MIP-1β was significantly lower at 21 days as compared to the 24 hours (p <0.05). IL-17, MCP-1, MIP-1β and TNF-α showed greater levels at 24 hours as compared to the corresponding baseline scores. 

## DISCUSSION

Invisalign aligners are an alternative treatment that provides greater aesthetics and more comfort for orthodontic patients.^9^ Thus, the demand for this type of device has significantly increased in the recent years; however, studies related to the biology of movement using aligners are scarce.

When assessing cytokine concentration levels during the study period, all mediators, except MIP-1β, showed fluctuations over time, with no statistically significant differences.

MIP-1β, which is considered a chemoattractant cytokine, is related to the activation and recruitment of monocyte/macrophage cell lines. Furthermore, this chemokine may play an important role in host response by recruiting inflammatory cells into active inflammation and inducing the release of other cellular mediators.^10^ Proinflammatory cytokines, such as IL-1, IL-6, and TNF-α, and resident cells, such as fibroblasts and osteoblasts, appear to induce chemokines such as MIP-1β.^11^ Thus, the role of chemokines in orthodontic tooth movement may be associated with the fact that these mediators are involved in the migration of monocytes to the periodontal ligament, where they will differentiate into osteoclasts and macrophages, essential cells for orthodontic movement.

In this study, the expression levels of MIP-1β were statistically different at T24h and T21d. This finding is contrary to that of Capelli et al,^12^ who showed a consistency in the concentration of this cytokine, without statistically significant differences, during the distalization of canines with retraction loops. Thus, the greater level of MIP-1β at T24h would be consistent with the release of inflammatory markers in early hours after a exposure to orthodontic forces. 

The proinflammatory cytokines, such as TNF-α, IL-1β and IL-8, are related to the acute phase of inflammatory response associated with tooth movement; therefore, their expression levels are increased during the initial hours after orthodontic force application.^11^ This increase in TNF-α and IL-8 expression levels was observed in the first 24 h after the aligner was installed. These results are consistent with those of a previous study that used conventional orthodontic appliances, with the insertion of NiTi wires and mechanical distal movement of canine before or after dental alignment and leveling.^1^ ^,^ ^14^ ^-^ ^17^

In contrast, IL-1β expression levels showed fluctuations over time, which is consistent with the findings by Iwasaki et al,^18^ where a peak concentration of this cytokine was not characterized within the first 24 h. Castrofolio et al^19^ demonstrated that a peak concentration of IL-1β occurred 21 d after orthodontic activation with Invisalign.

IL-17, considered a pro-inflammatory cytokine, acts synergistically with other cytokines, such as IL-1, IL-6, TNF-α. In this study, the results showed no significant changes in the concentration levels of IL-17 over the observation period, similar to a previous study that applied intrusive forces on the premolar teeth wherein no statistically significant changes in IL-17 levels were observed.^20^

Despite its importance in bone remodeling as a pro-inflammatory cytokine, the production of GM-CSF during orthodontic movement remains unclear. In this study, fluctuations in results were observed with no statistically significant differences, which is consistent with the results of a study conducted on adult patients using forces for the distalization of canines.^21^ However, in the adolescent patients of this same study, concentration levels were higher in 24h;^21^ which was also identified after 4 h in a study using forces for canine distalization.^17^ In another study, GM-CSF could not be evaluated because its expression was below detection levels.^22^

MCP-1 has been described as chemokine that stimulates osteoclast formation and bone resorption. The present study observed no changes in its levels over time, and this was in agreement with previous studies^12^ ^,^ ^16^.

IL-7 mediator was identified at low expression levels at all times and there are no human studies in literature that focus on the response of IL-7 to orthodontic treatment.

Despite the possible influence of periodontal inflammation on the results of cytokine analysis in the GCF, since there is a possibility of increased secretion due to periodontal plaque related inflammation; the periodontal indices in the present study remained low and presented a decrease over the studied time.

A possible explanation for the present findings relates to the type of device used in this study: it is possible to remove the device for oral hygiene. In addition, the patients were submitted to oral hygiene control by providing them with brushing instructions throughout the study period. Thus, the changes observed are strongly associated with stimulation of tooth movement.

In this study, concentration levels of inflammatory mediators were analyzed and longitudinally identified in a single sample using immunoenzymatic multi-analysis assay with microspheres. This assay has the ability to simultaneously measure a large number of targets with a small sample, thereby resulting in higher yields and lower costs.^23^

Considering the wide range of biological responses and factors interfering with GCF, the reduced number of participants may be considered a limitation of the present study. However, this study analyzed a total of 180 sites and a large number of previous studies used similar sample sizes.^1^ ^,^ ^12^ ^-^ ^17^ ^,^ ^19^ ^-^ ^20^ ^,^ ^22^

Another important limitation was that GCF is a transudate that is used to remotely study phenomena that occur in the periodontal ligament.^1^ ^,^ ^21^ ^,^ ^24^ Thus, these results should be interpreted with caution.

Finally, the present results warrant further investigations to clarify the biological mechanisms behind orthodontic aligners.

## CONCLUSIONS 

When using aligners (Invisalign), cytokine expression levels involved in cell recruitment indicates a role of these biomarkers in the tissue remodeling induced by light forces. Thus, further studies are needed to increase the knowledge regarding interferences and biological influences during orthodontic treatment with Invisalign. 
